# Cryopreserved Reticulocytes Derived from Hematopoietic Stem Cells Can Be Invaded by Cryopreserved *Plasmodium vivax* Isolates

**DOI:** 10.1371/journal.pone.0040798

**Published:** 2012-07-23

**Authors:** Florian Noulin, Céline Borlon, Peter van den Eede, Luc Boel, Catherine M. Verfaillie, Umberto D’Alessandro, Annette Erhart

**Affiliations:** 1 Unit of Malariology, Institute of Tropical Medicine Antwerp, Belgium; 2 Unit of Immunology, Institute of Tropical Medicine Antwerp, Belgium; 3 Stem cells institute, Catholic University Leuven, Belgium; 4 Medical Research Council Unit, Fajara, The Gambia; Walter & Eliza Hall Institute, Australia

## Abstract

The development of a system for the continuous culture of *Plasmodium vivax in vitro* would benefit from the use of reticulocytes derived from differentiated hematopoietic stem cells (HCS). At present, the need to use both fresh reticulocytes and fresh *P. vivax* isolates represents a major obstacle towards this goal, particularly for laboratories located in non-endemic countries. Here, we describe a new method for the cryopreservation of HSC-derived reticulocytes to be used for both *P. falciparum* and *P. vivax* invasion tests. Cryopreserved *P. falciparum* and *P. vivax* isolates could invade both fresh and cryopreserved HSC-derived reticulocytes with similar efficiency. This new technique allows the storage of HSC-derived reticulocytes which can be used for later invasion tests and represents an important step towards the establishment of a continuous *P. vivax* culture.

## Introduction

Outside sub-Saharan Africa, *Plasmodium vivax* is the most common human plasmodium species [1]. Though it has long been considered as less dangerous than *P. falciparum*, severe and fatal *P. vivax* cases as well as drug resistant parasites are now increasingly reported [2], [3], [4], [5]. Understanding of the biology and the transmission dynamics of *P. vivax* lags behind that of *P. falciparum*, partly because the maintenance of a continuous line of this parasite *in vitro* has not been established. The availability of an *in vitro* culture system would improved our understanding of the biology of *P. vivax*, e.g. invasion and relapse mechanisms, resistance markers, and would contribute to the development of new treatments and vaccines.

*P. vivax* preferentially invades reticulocytes [6] and thus in order to achieve a continuous *in vitro* culture system the availability of large amounts of these young red blood cells, which circulate in the peripheral blood at low concentration (1% of total red blood cells) and for a very short time (24 h), is required. Russell *et al.* [7] recently published a reliable invasion assay protocol using fresh *P. vivax* schizonts from fresh clinical isolates and enriched cord blood reticulocytes. It has been previously shown that reticulocytes can also be successfully produced through the differentiation of hematopoietic stem cells (HSC) [8] and that such HSC-derived reticulocytes may be used for *P. vivax* culture, though both the reticulocyte production and the parasite densities obtained were extremely low [9]. The contribution of this paper is to report an improved method to produce and cryopreserve HSC-derived reticulocytes to be later invaded by *P. vivax*, allowing research on *P. vivax* culture to be carried out outside endemic areas, increasing the number of teams potentially working on this subject and hence the chances for major discoveries.

## Materials and Methods

### Ethics Statement

***P. vivax*** **sample collection:** MUTM 2008-15 from the ethics Committee of the faculty of Tropical Medicine, Mahidol University, Bangkok, Thailand.

**Cord blood sample collection:** blood was collected anonymously and patients were informed orally with a possibility of opting-out. Each Patient was notified on the hospital admission form of this opting-out possibility.

Procedure was accepted by ethic committee of UZA and ITM.

Study was approved by the ITM review board, number: SBB.219.2007/1410.

### Hematopoietic Stem Cell (HSC) Isolation

Umbilical cord blood samples (40 ml each) were collected from pregnant women delivering at the University hospital, Antwerp (UZA), after obtaining an individual informed consent. Mononuclear cells (MNC) were separated by Ficoll-Isopaque (GE Healthcare) centrifugation (250 g, 10 min) and enriched for CD34^+^ cells by supermagnetic microbead selection using Mini-MACS columns (Miltenyi Biotech) according to a previously published procedure [10].

### HSC Culture

The amplification procedure was adapted from a three-step expansion of CD34^+^ cells by sequential supply of the culture with specific combination of cytokines and growth factors [11]. HSCs isolated from cord blood were cultured at 37°C, 5% CO_2_ in a modified serum-free media (IMDM, Biochrom) supplemented with L- Glutamine (4 mM, Sigma), Penicilline/Streptomycine (1%, Invitrogen), Inositol (40 µg/ml, Sigma), Folic acid (10 µg/ml, Sigma), Monothioglycerol (1.6 10^−4^ M, Sigma), Transferrin (120 µg/ml, Sigma), insulin (10 µg/ml, Sigma), Bovine Serum Albumin detoxified by beads resin AG501-X8 (Biorad) (BSA, 100 mg/ml, PAA) [12].

**Step 1 (day 0 to 8):** CD34^+^ cells were cultured with Stem Cell Factor (SCF, 100 ng/ml, Bioke), IL-3 (5 ng/ml, R&D System), Hydrocortisone (HDS, 10^−6^ M, Sigma), and Erythropoietin (EPO, 3 IU/ml, R&D System). At day 4, cells were diluted 1∶2 in IMDM medium completed with all the four above mentioned growth factors and incubated at 37°C for 4 additional days. At day 7, cells were re-suspended (10^6^ cells per vial) in culture medium (IMDM) and an equal volume of 80% Foetal Calf Serum (FCS)/20% DMSO solution was added drop by drop to obtain an IMDM/40% FCS/10% DMSO solution before progressively freezing them at −80°C using a Mr Frosty [13].

**Step 2 (day 8–11):** at day 8, 250 000 cells were added to each 25 cm^2^ flask and incubated in 5 ml of IMDM medium supplemented with only EPO (3 IU/mL).

**Step 3 (day 11–20):** the culture was maintained in IMDM without growth factors or cytokines and the medium was changed every 3 days. The culture was stopped at day 14 corresponding to the peak of reticulocytes counts determined by microscopic examination of thin films done by cytospin (Thermo scientific): 200 000 cells were washed with PBS once and re-suspended in 50 µL of PBS. 50 µL of Cresyl Blue(Merck)(previously diluted 1/100) were added to the tube and incubated 30 minutes. FCS (30 µL) was added to protect cells during cytospin centrifugation and the cells were placed in a cytospin funnel. After a centrifugation (700 rpm, 3 minutes), slides were removed from the funnel and stained with Giemsa. A reticulocyte scoring positive would contain two or more blue-stained RNA granules.

### HSC Cryopreservation

HSC derived reticulocytes at day 14 in the culture were subjected to three different cryopreservation protocols:

**Glycerolyte solution** [**14**] **:** 100 µL of Glycerolyte (Baxter) were drawn up to the cell pellet using an insulin syringe. Firstly, 20% of the volume of Glycerolyte was added to the blood cell suspension drop by drop while continuously agitating the tube to mix the content. This suspension was incubated 5 minutes at room temperature before adding the rest of Glycerolyte. RBC-Glycerolyte mixture was then aliquoted into cryovials and frozen at −80°C overnight before being stored in liquid nitrogen.

**Glycerol & Sorbitol solution** [**15**] **:** Cells were frozen in a medium composed of 28% glycerol, 3% sorbitol, and 0,9% NaCl diluted in distillate water. This solution was added drop by drop to packed cells, at room temperature. Cells were immediately transferred at −80°C.

**IMDM/10% DMSO/40% FCS solution** [**16**] **:** Cells were frozen according to a standard method with medium containing 10% DMSO/40% FCS as described above for the HSC culture at day 7.

Reticulocytes were kept in liquid nitrogen up to 1 year. Cells were thawed following the same protocol as for *Plasmodium falciparum*: the volume (V) of the thawed content was measured and transferred to a 50 ml centrifuge tube. Then 0.1 V of pre-warmed (37°C) 12% NaCl solution was added drop by drop to the parasitized red blood cells (pRBC), which were allowed to stay for 5 min at room temperature. Subsequently 10 V of 1.6% NaCl solution (pre-warmed at 37°C) were carefully added to the cells, which were then centrifuged for 10 min at 650 g without applying a break. The supernatant was discarded and 10 ml of 0.9% NaCl solution pre-warmed at 37°C was carefully added to the pellet, followed by a 5 min centrifugation at 650 g [17]. The cryopreservation protocols were evaluated according to the following criteria: viability of the cells after thawing, preservation of the reticulocyte population, presence or absence of clots, and presence of invaded reticulocytes by *Plasmodium*.

### FACS Analysis

HSCs cultures were analysed by flow cytometer at day 8, 11, 14 and 17 of their in-vitro maturation. After thawing the cryopreserved reticulocytes they were analysed by FACS, to assess the expression of stage specific markers: CD36, CD45, CD71 and CD235a. Briefly, 200 000 cells were centrifuged for 5 min at 650 g. The pellet was re-suspended in 100 µL of PBS buffer. 5 µl of CD45-PE (BD bioscience), 10 µL of CD71-APC (BD bioscience), 5 µL of CD36-FITC (BD bioscience), and 5 µL of CD235-PerCP (BD bioscience), were added to the cells and incubated for 15 min at room temperature in the dark. One tube containing only antibody isotypes coupled to fluorochromes was used as a negative control: 10 µL of IgG1γ-PE (BD bioscience), 5 µL of IgM-FITC (BD bioscience), 5 µL of IgG1 K isotype-PerCP (BD bioscience), and 5 µL of IgG1 K isotype-APC (BD bioscience). After incubation cells were washed with FACS buffer (Miltenyi Biotech) and re-suspended in 500 µL of buffer before analysis. FACS analysis were carried out on a FACScalibur 4 color cytometer (BD bioscience).

### Hemoglobin Content

The haemoglobin (Hb) content (foetal or adult haemoglobin) of the HSC derived reticulocytes was analyzed using the Kleihauer method [18]. Umbilical cord blood (≥95% of foetal Hb) and adult peripheral blood (≥95% of adult Hb ) were used as controls. Cells containing foetal haemoglobin will be stained in red whereas cells containing adult haemoglobin will look empty and only their membranes will be observed.

### Plasmodium Falciparum

*P. falciparum* D10 strains, cultured and cryopreserved in the Institute of Tropical Medicine, Antwerp, Belgium, (ITMA) cryobank were used for this study. Parasite isolates were thawed in a 37°C incubator until no ice was left in the tube. The thawing protocol was the same as the one used for reticulocytes. To check for the invasion of new RBCs (RBC derived from HSC maturation) by P. falciparum, pRBC were concentrated on a Percoll gradient (90%, 70%, and 40%; Sigma) [19] and centrifuged for 30 min at 2600 g without applying a break. After centrifugation, the layer between the 70% and 40% Percoll was kept, transferred into a new tube and washed with RPMI (Lonza). 1 µl of purified pRBC (≥90% mature forms) was mixed with 4 µL of the HSCs derived reticulocytes and 94 µL of RPMI medium supplemented with HEPES (25 mM, Lonza), L-glutamine (1 mM), gentamycin (40 µg/ml, Sigma), D-glucose (2%, Sigma), hypoxanthine (50 mg/L, Sigma) diluted in NaOH 1 M and 10% heat inactivated Human Serum (final volume: 100 µL, haematocrit 5%). The culture was incubated in a hermetic chamber flushed gas (90% N2, 5% CO2, 5% O_2_) at 37°C for 24 h. Parasite invasion was determined by microscopic examination of Giemsa stained thin films. The number of newly invaded cells by *P. falciparum* ring stages were counted and divided by the total number of cells to obtain the parasite density (% of invaded RBC). For each experiment, two technicians independently examined the slides and the mean parasite density was taken as the final value. If reading results were discordant (parasite counting difference ≥30%), both technicians re-read the slide until an agreement was found.

### Plasmodium Vivax

*P. vivax* isolates were collected from patients with acute *P. vivax* malaria (mono-infection with a density of >1 parasite/1000 red blood cells) attending the clinics of the Shoklo Malaria Research Unit (SMRU, Mae Sot, North-West Thailand), after obtaining their individual written informed consent. *P. vivax* isolates contained more than 80% early ring forms. White blood cells were removed after filtration on CF11 column (Whatmann) [20]; Parasites were frozen in Glycerolyte solution [21], as described above, and then transferred and kept in liquid nitrogen at the ITMA. Subsequently, *P. vivax* isolates were thawed following the same protocol as described above for *P. falciparum*, and were re-suspended in pre-warmed 37°C McCoy’s 5A (Gibco) medium supplemented with 20% heat inactivated human serum from normal AB group donors and 0.5% D-glucose. The medium containing *P. vivax* was then transferred to a 25 cm^2^ culture flask, flushed with gas (90% N2, 5% CO2, 5% O_2_), and placed in a 37°C incubator for 36 to 44 hours to allow the maturation into schizonts. Parasite stage was determined by microscopic examination of Giemsa stained thick and thin films. Mature schizonts were concentrated in 45% Percoll by centrifugation for 15 minutes at 1580 g without applying a break (Sigma) [22]. Purified schizonts (≥90% mature forms) were mixed with HSCs derived reticulocytes resulting in a starting parasite density between 0.5 and 1%. Complete Mc Coy’s medium was added in order to obtain a 5% haematocrit level and the culture was incubated at 37°C in a hermetic chamber flushed for 24 h with gas (90% N_2_, 5% CO_2_, 5% O_2_) [7]. The efficiency of invasion was checked as described above for *P. falciparum.*

**Figure 1:**
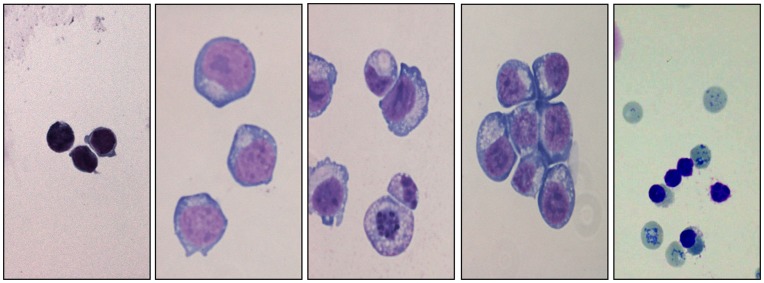
Production of reticulocytes from HSCs, morphologic analysis after Giemsa staining. (A) Human CD34+ cells isolated from cord blood (B) Day 4 of culture, (C) Day 8- (D) Day 11- (E) Day 14 of culture, reticulocytes are highlighted by cresyl blue staining (arrows).

**Figure 2:**
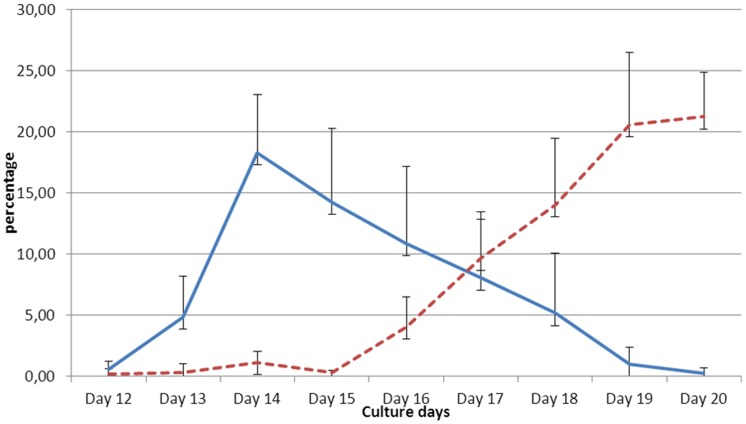
Evolution of reticulocytes (continuous line) and red blood cells (dotted line) counts from day 12 to day 20 of HSCs maturation. Each value represents the median a total of 6 independent experiments, and vertical bars represent the interquartile range (IQR). Reticulocytes were identified by cresyl blue staining.

### Data Analysis

During HSC maturation, reticulocytes and RBC were counted for each selected day during the maturation process. Median value and interquartile range (IQR) were calculated for the reticulocytes counts (6 independent experiments) as well as for the expression of the surface markers measured by FACS (3 independent experiments). Parasite densities for *P. vivax* invasion assays were calculated per 500 counted cells. The number of infected cells was divided by the total number of counted cells and multiplied by 100 to obtain a percentage.

## Results

### Amplification of Erythroid Cells

Microscopic examination of thin films of the erythroid cells (Figure 1) confirmed a normal differentiation pattern and the commitment of the HSC culture to the erythroid lineage as defined elsewhere [23]. A total of 30 independent experiments were successfully carried out, and median reticulocyte and RBC population counts were computed at regular intervals (every 3 days between day 8 and day 20) for 6 experiments (Figure 2). The first reticulocytes were observed at day 12 of differentiation, and their number dramatically increased between days 13 and 14 (from 5% to 18%), corresponding to a peak of enucleation. Subsequently, the percentage of reticulocytes decreased steadily (10% at day 16) and almost disappeared at day 19 of differentiation. The haemoglobin content measured at day 14 of maturation was mainly foetal (about 90%, data not shown). Mature red blood cells (RBC) showed an opposite trend as compared to reticulocytes as they started to increase following the reticulocyte peak (0.4% at day 15), outnumbered the reticulocytes at day 17 and reached a plateau of above 20% at day 20. After cryopreservation, the cells viability was up to 70% and the proportion of reticulocytes remained stable compared to the one before freezing (except for glycerolyte which was not tested due to the presence of clots during the thawing process).

**Figure 3:**
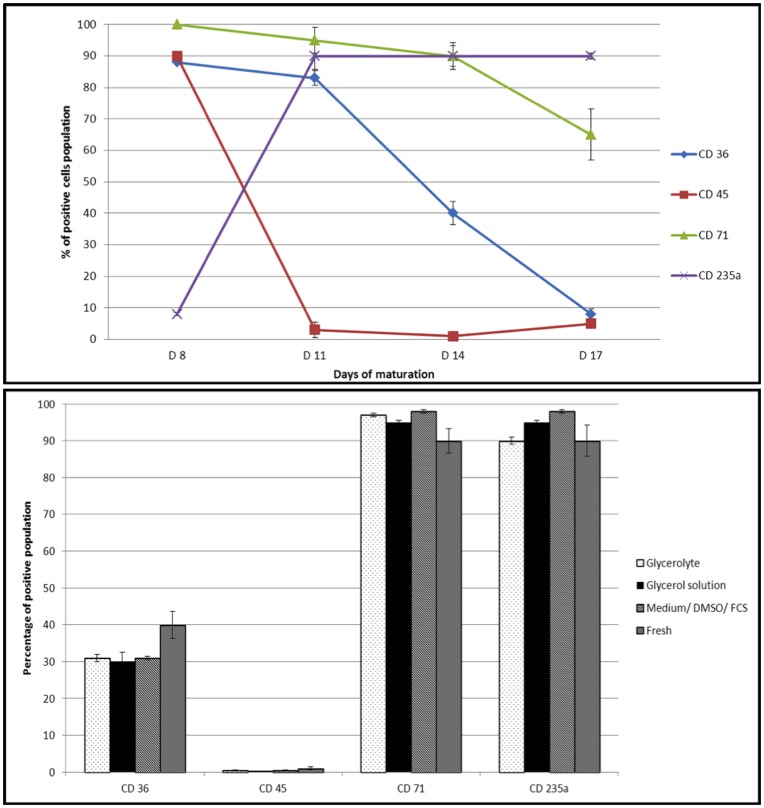
Expression of reticulocyte surface markers by flow cytometry. 3a: Expression of HSCs surface markers at different maturation stages characterized by flow cytometry. Cells were collected, respectively at 8 days, 11 days, 14 days and 17 days of maturation and stained with specific antibodies against CD36, CD45, CD71 and CD325a receptors, respectively. Each value represents the median percentage for 3 independent experiments (vertical bars represent the extreme values in this case). 3b: Comparison of the surface markers expression (%) at day 14 after thawing for 3 different cryopreservation protocols compared to fresh HSC derived reticulocytes (median % values of 3 independent experiments; vertical bars represent the extreme values in this case).

### FACS Analysis of HSC Maturation

The proportion of positive cells expressing different surface markers was calculated at different time points (Figure 3a). The CD45, a specific marker for young cells (pre-proerythroblast stage), decreased dramatically from 90% at day 8 to 3% at day 11, while CD235a (Glycophorin A marker) followed the opposite trend. Both CD71 and CD36 (both markers of stages between the proerytroblast and mature RBC) were highly expressed at day 8, 100% and 80% respectively, and decreased following a different pattern until day 17: the CD71 decreased slightly to 65% at day 17, while the CD36 dramatically decreased to less than 10% at day 17. A freezing cycle (up to 1 year in the frame of this work) of the HSC derived reticulocytes which were matured till day 14 did not affect the expression of specific reticulocytes markers for any of the three protocols tested. Indeed the comparison with the freshly produced HSC derived reticulocytes gave similar results: (Figure 3b) a high expression of CD71 (between 95% and 98% according to the freezing protocol *versus* 90% for fresh reticulocytes at day 14), and CD235a (between 90 and 98% *versus* 90% in fresh reticulocytes), a quasi-absence of CD45 (around 0.5% *versus* 1%), and the expression of CD36 reaching 30% for the 3 protocols tested (*versus* 40%).

### Invasion Assays

**Figure 4:**
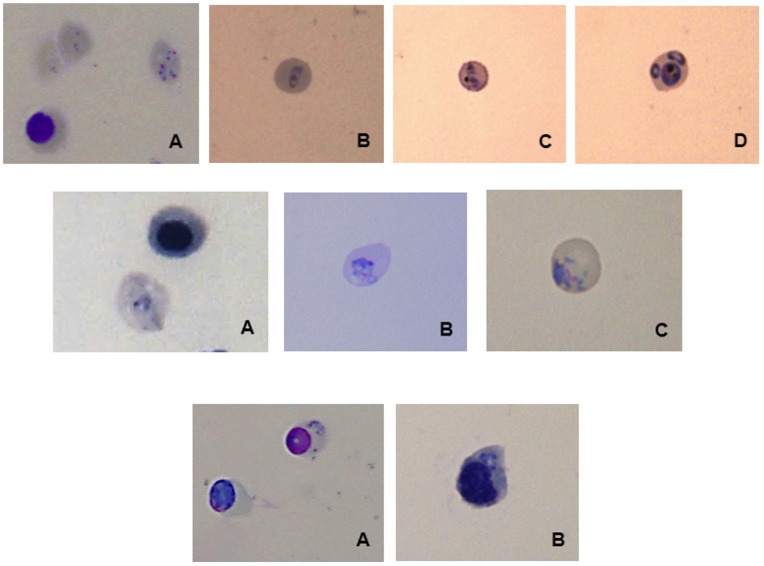
Pictures of HSC-derived reticulocyte invaded by *Plasmodium*. 4a: Infected enucleated cells by *P. falciparum* 24 h after invasion. (A) Fresh reticulocytes derived from HSCs maturation, (B) Cryopreserved reticulocytes with glycerolyte, (C) Cryopreserved reticulocytes with glycerol solution, (D) Cryopreserved reticulocytes with Medium/40% FCS/10% DMSO (3 replicates). 4b: Infected reticulocytes by *P. vivax* 24 h after invasion. (A) Fresh reticulocytes derived from HSCs maturation, (B) Cryopreserved reticulocytes with glycerol solution, (C) Cryopreserved reticulocytes with Medium/40% FCS/10% DMSO (8 replicates). 4c: Infected erythroblast (A) By *P. falciparum*. (B) By *P. vivax* (8 replicates).

**Figure 5:**
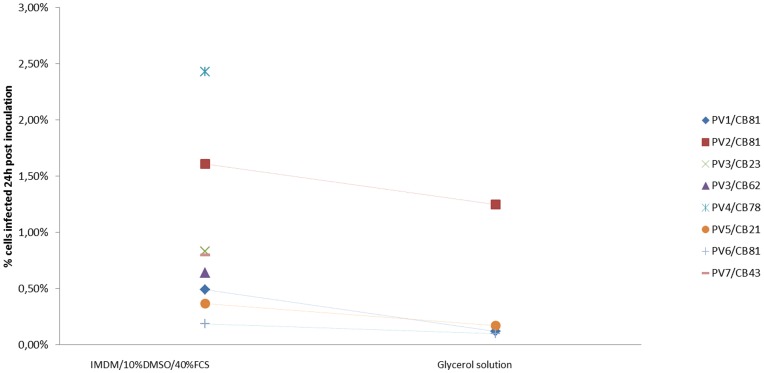
Parasitemia of infected HSC derived cells for the 2 protocols of cryopreservation (Medium/10%DMSO/40%FCS and Glycerol solution) 24 h after invasion assay with *P. vivax* cryopreserved isolates.

Both *P. falciparum* and *P. vivax* could successfully invade freshly HSC derived reticulocytes (Figure 4a and 4b). Parasite density after invasion of fresh reticulocytes was around 0.5% for *P. vivax* and 4% for *P. falciparum*. Parasite invasion was then tested on the cryopreserved reticulocytes derived from HSC maturation. *P.* *falciparum* could invade cryopreserved reticulocytes subjected to any of the 3 cryopreservation methods (parasite density: 4.2% for glycerol solution, 4.1% for Glycerolyte and 8% for the IMDM/10% DMSO/40% FCS; 3 independent assays). However, the method using the Glycerolyte solution was discarded as clots were observed during the thawing process. Therefore, for *P. vivax* invasion, only reticulocytes cryopreserved with the Glycerol solution and the Medium/10% DMSO/40% FCS solutions were used. These were successfully invaded with the identification of ring stages 24 h post invasion and the parasite density was counted for all 8 independent experiments (Figure 5). *P. falciparum* and *P. vivax* could invade the erythroblasts stages, with both fresh and cryopreserved HSC derived cells (Figure 4c).

Parasite density obtained after invasion of cryopreserved HSC derived reticulocytes varied substantially (from 0.19% to 2.43%) according to the *P. vivax* isolate used. However, parasite density for each single parasite isolate did not vary when using different batches of cryopreserved reticulocytes. The range of parasite densities after invasion was between 0.19% and 2.43% for reticulocyte cryopreserved in the IMDM/10% DMSO/40% FCS solution. In comparison, invasion rate of reticulocytes cryopreserved in glycerol solution was 30 to 50% less efficient in 4 replicates) (Figure 5).

## Discussion

This is the first report showing a reliable cryopreservation protocol for HSC-derived reticulocytes derived that can be successfully invaded by both cryopreserved *P. falciparum* and *P. vivax* isolates. This will contribute to the establishment of a continuous *P. vivax* culture, since the latter requires a continuous supply of reticulocytes in a large amount. Until now, availability of adequate quantities of reticulocytes was a bottleneck, as they represent only 1% of all circulating cells in the blood stream [24]. Several approaches have been tested to tackle this problem. One used blood from hematochromatosis patients [25] as they need to be regularly bled and have a higher percentage of reticulocytes. This method allowed for short-term *P. vivax in vitro* cultures for up to 15 days. However, the limited access to hemochromatosis patients is a drawback. A second source of reticulocytes is cord blood, in which the reticulocyte concentration is slightly higher (3–8%) than in adult blood; this source has been previously used permitted maintaining *P. vivax* fresh isolates in culture for about one month [26]. However, the low amount of reticulocytes obtained did not allow for an optimal parasite multiplication (parasite density <0.7%). Moreover, substantial variation of parasite densities obtained makes this protocol unsuitable for studying *P. vivax* biology.

Cord blood HSCs represent a valuable source, as reticulocytes can be produced in substantial quantities [9], [27]. The differentiation protocol presented here was adapted from the one published by Giarratana *et al.* [8], [28], which originally matured HSCs into erythroid lineage by using a co-culture with stromal cells. The latter are mainly used to retain nucleated cells, free nuclei and debris so that the culture appears cleaner. We have modified this technique by omitting the co-culture with stromal cells, which resulted in a less pure population of enucleated cells in our model (20% enucleated/80% nucleated cells *versus* 100% enucleated cells when using stromal cells) since the nucleated cells were not retained by the MS5 layer. Nevertheless, while Giarratana aimed at obtaining RBCs with a lifespan of about 120 days, achieved during the later stages of RBC maturation, our purpose was to obtain reticulocytes whose lifespan is 2 days. This explains why maturation was stopped at the peak of the reticulocyte concentration (D14). Moreover, reticulocytes obtained after maturation on a MS5 layer gave similar result in term of invasion than those produced without stromal cells (data not shown). A previous attempt of producing HSC-derived reticulocytes without using stromal cells in co-culture attained a maximum reticulocyte concentration of 0.5% [9]. This is in contrast with the results of our methods which produced a 40-fold (20%) higher concentration of reticulocytes. The low efficiency of the previous method could be explained by several factors such as the use of non-detoxified BSA (a key factor for the last steps of maturation and enucleation) [27], a very high (lethal?) concentration of monothioglycerol, or the absence of inositol or folic acid. It is also important to mention that the parasite density in culture supported by HSC-derived reticulocytes obtained with our method was substantially higher (2.5% vs 0.0015%) than that reported for the previous one [9], providing sufficient biological material to possibly keep the culture for a longer period.

During the invasion assays we performed, the cell population was not purely composed by reticulocytes but contained also erythroblasts. Both *P. falciparum* and *P. vivax* were able to invade these cells, as previously reported [29]. No information on the quality of the parasite development in this type of cells is currently available.

FACS analyses of both fresh and cryopreserved reticulocytes showed the expression of CD235a marker (Glycophorin A), an important receptor for *P. falciparum* invasion [30]. Its role in *P. vivax* invasion has not been described yet but its homology with the Duffy Binding Protein (DBP) [31], [32] suggests a potential role in the *P. vivax* invasion process of the reticulocytes. Thus the cryopreservation protocol kept the important receptors on the reticulocyte membrane.

The added value of the work presented is the set up of a cryopreservation technique for the long-term storage of cord blood HSC-derived reticulocytes, providing a continuous source for the performance of invasion assays and possibly the maintenance of a *P. vivax in vitro* culture. The technique is robust as it has been tested on several HSCs batches of that gave comparable results with a single *P. vivax* isolates (Fig.5, PV3/CB 81 and 23). The reticulocyte cryopreservation using Glycerolyte was abandoned due to the presence of clots during thawing. It is unclear why this occurred but a possible hypothesis would be the harmful effect of glycerolyte on nucleated cells.

The source of reticulocytes for a *P. vivax* invasion assay is not limited to HSC-derived cells. Borlon *et al* [32] were able to maintain a short-term (up to 10 days) *P. vivax* culture using reticulocytes directly concentrated from cord blood. This was done with similar efficacy with both fresh and frozen parasites and reticulocytes (using Glycerolyte 57 as cryopreservant). The advantage of concentrating reticulocytes from cord blood is that these are almost immediately available (pending availability of cord blood) while HSC-derived ones need 14 days to mature. Nevertheless, with the former technique the concentration process (percoll centrifugation, application of several washing/concentration steps) could damage the cells and affect the invasion efficiency while using HSC-derived reticulocytes may guarantee a more homogeneous and standardised cell population. Eventually, using both methods: concentrated from cord blood and HSC-derived reticulocytes could substantially increase the amount of available reticulocytes from a single cord blood sample.

In conclusion, the cryopreservation method described in this paper provides a continuous and substantial source of reticulocytes, possibly a first step to achieve the continuous *in vitro* culture of *P. vivax*. In addition, the possibility of cryopreserving both reticulocytes and parasites offers the opportunity of investigating *P. vivax* invasion assays/culture in non-endemic countries, opening this field of research to more research groups thus increasing the probability of fast advances in knowledge.
